# Building trust in the age of human-machine interaction: insights, challenges, and future directions

**DOI:** 10.3389/frobt.2025.1535082

**Published:** 2025-07-14

**Authors:** Sakshi Chauhan, Shashank Kapoor, Malika Nagpal, Gitanshu Choudhary, Varun Dutt

**Affiliations:** Applied Cognitive Science Laboratory, Indian Knowledge System and Mental Health Applications Centre, Indian Institute of Technology Mandi, Mandi, India

**Keywords:** trust in human-machine interaction (HMI), explainable artificial intelligence (XAI), human-robot interaction (HRI), behavioral change, cross-cultural trust dynamics, psycho-social factors in AI

## 1 Introduction

Trust is a foundation for human relationships, facilitating cooperation, collaboration, and social solidarity (Kramer, 1999). Trust in human relationships is generally based on factors like dependability, competence, generosity, and sincerity (Mayer et al., 1995; Lewicki and Bunker, 1996). Social norms, emotional intelligence, and the power of forecasting others’ behaviors help create shared knowledge and mutual respect (Coleman, 1990; Rotter, 1980).

As technology more and more becomes incorporated into everyday life, especially by means of artificial intelligence (AI) and robotics, the concept of trust has shifted paradigmatically (Lankton et al., 2015). In Human-Robot Interaction (HRI), trust does not derive from emotional familiarity or social intuition but rather from properties of the system itself, such as functionality, transparency, and predictability (Hancock et al., 2011). This invokes basic questions: Can humans ever trust machines? If they can, how is that trust established, sustained, or dissolved?

There is growing evidence that humans can work with robots in various situations, such as search-and-rescue missions, education, and healthcare (Breazeal, 2003; Chen and Barnes, 2014; Nagpal et al., 2024; Nandanwar and Dutt, 2023). For instance, latest research utilizing Proximal Policy Optimization (PPO) and Generative Adversarial Imitation Learning (GAIL) identify that robots have the ability to excel over human peers in difficult search-and-retrieve tasks in a situation where trust is calibrated (Kapoor et al., 2024a; 2024b). In the same vein, emotionally responsive robots have demonstrated potential in improving language learning achievement in school children (Nagpal et al., 2024), whereas affective conversational agents assist in stress and anxiety reduction in patients (Nandanwar and Dutt, 2023).

But embedding AI systems within fields such as autonomous driving, military action, and healthcare introduces novel trust challenges. These are the opacity of decision-making by algorithms, variable levels of autonomy, and cultural compatibility clashes in user expectations (Chen and Barnes, 2014; Goodall, 2014; Schaefer et al., 2016). Even when AI is reliable, a lack of explainability will undermine user trust. Consequently, Explainable AI (XAI) is essential in closing the cognitive and affective space between humans and machines (Arrieta et al., 2020).

However, trust in HRI is not always built. It differs by cultural environment, personality type, and task context. Although tremendous strides have been made in the modeling of trust as a system performance function, current models tend to overlook dynamic, emotional, and socio-cultural aspects (Eiband et al., 2018; Hoff and Bashir, 2015).

This opinion paper contributes to the discussion by comparing the building blocks of trust in human-human and human-robot interaction. It presents the Trust-Affordance Adaptation Model (TAAM)—a theoretical framework that aligns trust-building tactics with domain requirements. We contend that emotional investment and functional openness need to be traded off depending on context, and we propose the incorporation of psycho-social cues, like biosensor information, into trust modeling. Through a synthesis of current literature and findings of recent empirical research, the paper provides a guide for developing reliable AI systems that are emotionally engaged, culturally adaptable, and context-sensitive.

## 2 Trust in human-human interaction

**FIGURE 1:**
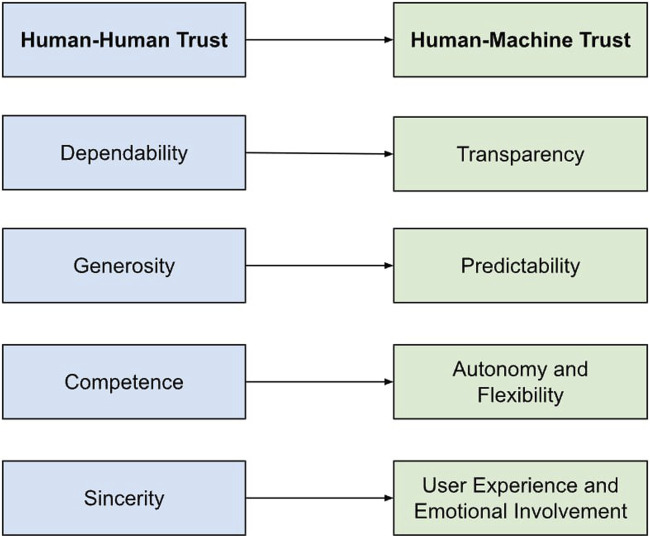
The primary elements of trust in human-machine relationships are transparency, predictability, autonomy and flexibility, user experience, and emotional engagement, while dependability, generosity, competence, and sincerity are the most significant elements in human-human relationships.

Several basic disciplines, such as organizational behavior, psychology, and sociology, have thoroughly researched the interpersonal trust phenomena (Lewis and Weigert, 1985; Rotter, 1980). Based on Figure 1, it appears that there are many basic factors to consider that facilitate or sustain relationship trust. Establishing and maintaining trust is particularly difficult in business settings. Dependability is the primary trait, especially in firms where cooperation and production rely on one another (Mayer et al., 1995; Dirks and Ferrin, 2002). When individuals working in a team possess the appropriate level of confidence in their competence, they are capable of cooperating and actually achieving shared objectives.

The correspondences between human-human trust and human-machine trust is shown in Figure 1. These correspondences are based on well-established theoretical constructs for trust in automation and HRI. For instance, ‘dependability’ of human-human trust equates with ‘transparency’ of human-machine trust because both express reliability of intentions and actions (Muir and Moray, 1996). ‘Generosity’ translates into ‘predictability’, expressing anticipation of regular behavior that meets user requirements (Hancock et al., 2011). ‘Competence’ is a robot’s ‘autonomy and flexibility’, which is its ability to accomplish tasks efficiently. ‘Sincerity’ parallels emotional involvement in robots, reflecting their perceived warmth and empathy in interactions (Nass and Moon, 2000). These parallels, illustrated in Figure 1, are conceptual and aimed at drawing meaningful bridges across social and technological domains of trust.

Generosity, which refers to someone caring for you, facilitates the creation of trustful networks (Mayer et al., 1995). The element of human empathy maintains safeguarding as well as mutual respect for each other which comprises the basics of any relationship. In other extremities, people are even more willing to cooperate with others who strive to help them (Dirks and Ferrin, 2002).

Another fundamental trait that brings about trust is competence, which is the ability to perform tasks in an effective manner with adequate resources which is particularly critical in the business arena (McAllister, 1995). In occupational groups, mutual trust between members and mutual trust in other members’ competence improves collaboration in group work along with decision making, thus improving productivity (McNeese et al., 2021).

Reciprocal trust can only be achieved through sincerity. One’s integrity embraces honesty and fairness, which considers an individual’s credibility and builds trust in the personal and organizational spheres (Mayer et al., 1995). Individual moral uniformity underwrites the founding base of trust. Furthermore, sincerity acts as the bedrock of moral relations that enhances cooperation and solidarity within a group.

## 3 Trust in human-robot interaction

As a component of HRI and like with all human interactions, trust is also an important factor that requires special attention. It is widely accepted that trust in systems is negatively impacted if there is a lack of system transparency or explainability (Hancock et al., 2011). Understanding what a robot is doing and how it arrives at its decisions influences trust, too. Explainable AI, or XAI, strives to make the reasoning behind automated systems’ decisions more understandable, which in turn enhances reliance and endorsement (Arrieta et al., 2020).

Human-robot collaboration studies put forward transparency as an important factor for trust. In other cases, simple but reliable robots exceed humans’ performance in PPO search and retrieval. It has been proposed that the formation of trust and collaboration is enhanced when robots meet expectations and provide comprehensive explanations regarding their decisions (Kapoor et al., 2024a; 2024b).

Another central dimension of HRI is predictability. Trust in robots, as with humans, relies on repetitive execution of the same tasks. It is a question of predictability: To what extent can the robot’s actions be anticipated? Dependable and consistent actions lead to trust, while erratic actions create suspicion (Hancock et al., 2011).

The levels of trust in human-robot interaction are notably impacted by autonomy and flexibility. Trust in robots is developed when there is an effective adaptation to drastic changes in the environment while still performing optimally (Beer et al., 2014). Nandanwar and Dutt (2023) explain that robots portraying emotions such as tension and anxiety build user trust. Therefore, in order to build trust, highly autonomous robots should respond to novel requests in a timely manner aligned with user expectations.

Furthermore, user experience and emotional engagement greatly impact trust in HRI positively. A robot’s emotive traits and emotionally evocative interactions can shape trust (Brave et al., 2005). This becomes important during nursing or companionship scenarios where forming emotional connections adds credibility to a robot’s actions (Breazeal, 2003). New studies show that emotionally responsive conversational robots can evaluate and mitigate adverse psychological states, support wellbeing, and create trust (Nandanwar and Dutt, 2023).

## 4 Comparative analysis: human-human *versus* human-robot trust

Despite some similarities, human-human trust and human-robot trust differ fundamentally at their core. The basis of trust among people stems from social ties or emotional connections (Lewis and Weigert, 1985), which is developed through experiences together and understanding one another (Rotter, 1980). Such interpersonal trust is usually boosted by ongoing interactions, which increases esteem and gratitude (Coleman, 1990).

On the other hand, trust in HRI derives from clarity and predictability associated to functional performance. Generally, people tend to trust robots or AI systems due to their dependable and efficient execution of tasks (Hancock et al., 2011). Robotic systems are deemed reliable when they meet specific performance targets and explain their operational state accurately. Unlike humans, who may forgive occasional lapses of unreliable behavior due to emotional connections that exist between them, robots build trust through consistent delivery of expected tasks (Kapoor et al., 2024a; b). Within the research involving PPO and GAIL on intricate search tasks using diverse robots, the need for reliability and transparency in trustable machine performance is emphasized (Kapoor et al., 2024a; b). In the context of robots, trust becomes more transactional as it is determined by whether expectations rather than being influenced by relationships cultivated. The study of trust in human-robot interaction (HRI) is based on observable behaviors and outcomes of robots or AIs (Hancock et al., 2011). For trust to be built, actions and performance must be clearly demonstrable. Human trust, however, is subject to strong emotional bonds and can overlook some lapses in reliability.

In the absence of affective history in HRI, even small failures by a robot can disproportionately reduce trust, supporting the need for real-time calibration of trust frameworks (Hoff and Bashir, 2015). For instance, healthcare or emergency response robots need to rise above passivity and actively detect user hesitation—providing explanations or reassurances for trust repair.

Trust among humans develops over time as a result of interaction, shared experiences, and maintained communication (Lewicki and Bunker, 1996). This characteristic means that it can evolve positively or negatively based on social interaction. Trust can be strengthened by positive experiences, however, it can also be reconstructed through communication and reconciliation during crises (McAllister, 1995).

Although trust concerning Human-Robot Interaction (HRI) may vary over time, at any specific moment it still relies upon the robot’s effectiveness or its clarity of communication (Hancock et al., 2011). People’s reliance on a robot’s capabilities in a certain field is contingent on how well the robot performs within a defined context. This type of variability requires that HRI be adjusted dynamically, which means that users need to evaluate the actions of the robot in real-time. In sensitive situations like healthcare and disaster response, adjusting levels of trust according to how well a robot performs is vital (Hoff and Bashir, 2015). Robots could best utilize biosignals like galvanic skin response or eye-tracking data to adjust to the user’s level of trust, which is a poorly developed area.

Different cultures can affect trust in diverse societies and in turn affect human-human interactions differently than human-robot interactions. In human interactions, trust is affected by the cultural practices of collectivism or individualism, which impact the perception of loyalty, transparency, and autonomy as trust-relevant traits (Gelfand et al., 2007). Furthermore, in HRI, culture affects how users perceive and engage with robots within a specific context. There are countries that will embrace autonomous systems while others may be very suspicious or even hostile towards them. For example, users from collectivist cultures may expect robots to demonstrate relational behaviors while individualist cultures expect more emphasis on autonomy and control (Li et al., 2010). Thus, there is a need for cross cultural study which designs robotic systems that adapt to different cultures using social context adaptable trust framework.

Although culture-specific trust structures have been described at a conceptual level, their translation into the real world is underdeveloped. Working practice might include culturally adaptive robot behavior, for example, adjusting verbal styles, proxemics, and interaction style according to background user. For instance, it has been indicated that Japanese users like robots that are humble and polite, but American users can like more assertive and autonomous robot behavior (Li et al., 2010; Rau et al., 2009). Robots can make their emotional expressiveness and engagement strategies more amenable to deeper trust building by incorporating culturally grounded preferences by training machine learning models on region-specific interaction data. Research in the future can try adaptive modules that tune robot attitude according to user nationality, linguistic orientation, or even religious traditions (Złotowski et al., 2015).

## 5 Discussion

The consideration of trust in human-robot interaction (HRI) has identified gaps that are critical for research and understanding how trust is built, sustained, and navigated within systems of HRI (Hancock et al., 2011). The impact AI explainability has on trust sets the starting point of a particular high-stakes domain. The urgency of the problem increases when it comes to defense, transportation, and healthcare (Miller, 2019). Users inevitably need to comprehend the rationale behind AI powered systems’ decisions that could drastically alter their circumstances. Trust can be greatly aided by mitigating the opacity of, and hence, the decision-making processes within AI systems (Arrieta et al., 2020). A promising area of future work is to design context-sensitive XAI models that have the ability to adjust modifications by changing the timing and detail of the explanation granted based on what the user requires.

The integration of psychosocial elements into trust frameworks for human-robot interaction is a promising new direction for research. Trust is heavily influenced by previous encounters, preconceptions (Sheridan et al., 2016), biases, personality traits, and sociocultural contexts (Hoff and Bashir, 2015). While the majority of computational models attempt to address some of these variables, they do so in an inadequate manner. As an example, trust models aimed at predicting trajectories of trust across diverse user groups, need to incorporate more psychosocial elements and behavioral as well as physiological sensing—like thermographic imaging or GSR—to better adapt to various user groups. A case in point is from education, where it has been demonstrated that emotionally adaptive robots can bolster student learning by increasing trust via emotional alignment (Nagpal et al., 2024).

For HRI systems, another equally important focus of research is trust recalibration in real time. Trust is always in need of new models that continuously gauge and adjust user trust in relation to evolving interactions and feedback loops (Schaefer et al., 2016). This becomes critical in more volatile settings like military operations or disaster response, where evaluation and readjustment of trust need to be incessantly done with respect to how well the robot is performing in the context of a constantly shifting environment (Chen and Barnes, 2014).

While biosensors like galvanic skin response (GSR), thermography, or eye-tracking hold promising streams for real-time trust estimation, a number of methodological issues remain. These encompass signal noise, context-dependency, individuality, and the difficulty of establishing physiological responses to targeted trust dimensions (Calvo and D’Mello, 2010; Nourbakhsh et al., 2017). The dynamic and multi-dimensional character of trust also renders it difficult to extract signal components that capture trust exclusively, as opposed to associated constructs such as stress or engagement. In addition, longitudinal calibration to account for individual baselines is commonly necessary, yet another obstacle to real-time use. Overcoming these shortfalls necessitates inter-disciplinary approaches that merge psychological profiling with adaptive sensor fusion and machine learning pipelines to manage noisy and incomplete data (Pfeifer et al., 2023).

Potential tools for calibrating trust may involve context-sensitive behavioral monitoring, real-time stress level assessment, or predictive algorithms for trust decay capable of enabling robots to preemptively engage in trust repair (Calvo and Peters, 2014). For instance, in healthcare, conversational AIs are able to identify symptoms of anxiety and depression, providing real-time language or tone modulation which highlights the recalibration of trust in sensitive settings (Nandanwar and Dutt, 2023).

To address these complexities, we propose the Trust-Affordance Adaptation Model (TAAM)—evolving a conceptual model which positions a domain’s specific affordances for trust building against its specific expectations. Unlike static models, TAAM posits that mechanisms of trust, such as emotional involvement, engagement, transparency, predictability, and personalization, are contextually relativized in their prominence. For example, in defense applications, trust may be rooted primarily in transparency and dependability, whereas in healthcare or education (Wagner et al., 2018), emotional engagement and adaptive personalization may prevail.

TAAM suggests that trust calibration needs to dynamically respond to context-dependent affordances. As an example, within a healthcare environment, a robot’s affective responsiveness and individualized feedback could be more influential to user trust than transparency with regards to its internal algorithms. A defense robot deployed in high-risk environments, however, would have to trade off predictability and transparency to establish human confidence in a matter of milliseconds. In educational contexts, emotionally intelligent avatars that adjust tone and body language have been found to enhance learning participation and trust (Nagpal et al., 2024). These instances demonstrate that trust affordances might be operationalized in varying ways based on domain-specific user emotional needs and expectancies.

**FIGURE 2:**
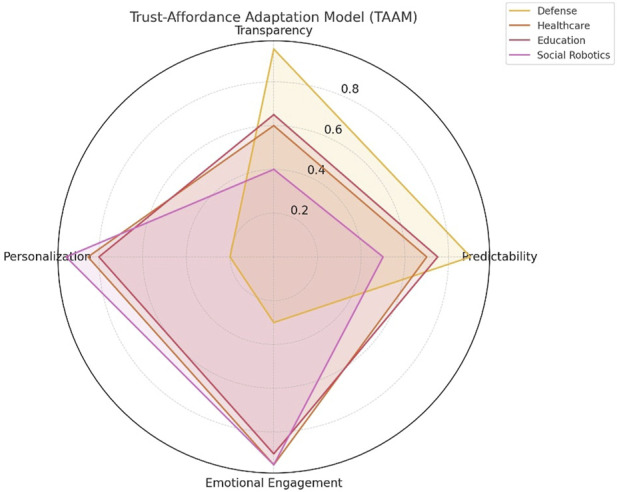
Conceptual radar chart showing the relative value of trust affordances—transparency, personalization, emotional engagement, and predictability—within four domains: defense, healthcare, education, and social robotics. The numeric values employed are hypothetical, not based on experimental data, and constitute domain-informed judgments aggregated from literature.

TAAM’s trust prioritization within specific domains is illustrated on the radar chart in Figure 2. This model advocates for the creation of AI systems which are functionally robust and equally contextually and emotionally intelligent. Through adding biosensors and feedback loops, culturally-informed models, TAAM paves the way for real-time personalized trust recalibration in human-robot interaction.

Values in Figure 2 are calculated based on our own integration of previous empirical and theoretical literature (e.g., Hancock et al., 2011; Kapoor et al., 2024a; Nandanwar and Dutt, 2023) and constitute relative importance of trust aspects within domains. For instance, transparency is paramount in defense environments, whereas emotional engagement prevails in education and social robotics. These domain-specific mappings serve to facilitate the TAAM model’s flexibility.

In conclusion, addressing the gaps in cross-cultural research regarding trust in robots from diverse parts of the world is critical. Culture, as Li et al. (2010) indicates, is an important factor that influences trust and how users robotic systems. Some cultures may accept autonomous robots as efficient partners, while others may not accept or embrace them. It is through cross-cultural research that these differences may be discovered and used to automate robots that respect local customs and values. Culturally sensitive robotic systems as Gelfand et al. (2007) outlines, is what may likely ensure social trust and acceptance for the global deployment of robotic systems.
